# Is Low-Dose Lung Radiotherapy for Severe COVID-19 Pneumonia a Therapeutic Alternative?

**DOI:** 10.7759/cureus.65033

**Published:** 2024-07-21

**Authors:** Fatima Safini, Bouchra Amaoui, Kenza Oqbani, Sanae Abbaoui, Slimane Semghouli

**Affiliations:** 1 Department of Radiation Therapy, Souss-Massa University Hospital, Agadir, MAR; 2 Faculty of Medicine and Pharmacy, Ibn Zohr University, Agadir, MAR; 3 Medical Physics, Higher Institute of Nursing Professions and Health Techniques, Agadir, MAR

**Keywords:** low dose, sars-cov-2, lung radiotherapy, pneumonia, covid-19

## Abstract

Low-dose radiotherapy (LD-RT) has historically been used to treat severe bacterial and viral pneumonia due to its immunomodulatory properties. However, this technique has been delayed since the advent of antibiotics and the carcinogenic risk associated with radiation therapy. Currently, it represents an alternative therapeutic in the SARS-CoV-2 pandemic which leads sometimes to fatal pneumonia, especially given the limited availability of effective treatments for severe forms of pneumonia. Numerous in vitro and in vivo studies have demonstrated the anti-inflammatory effect of LD-RT and have reported clinical and biological improvement in patients with severe pneumonia. In this literature review, we report the data and results of various published clinical trials.

## Introduction and background

The COVID-19 pandemic is one of the greatest challenges facing healthcare professionals worldwide. Severe acute respiratory syndrome represents the first cause of death from COVID-19. According to the WHO data published in 2024, the total number of deaths directly or indirectly associated with the COVID-19 pandemic, from its beginning until December 2023, was about 6,991,842. The number of new cases of COVID-19 and deaths has declined significantly since the introduction of vaccines [1].

Several therapies have been tested in the management of the severe form of COVID-19 pneumonia, such as hydroxychloroquine, antivirals (lopinavir/ritonavir, remdesivir), and interferon. However, they have not shown significant effectiveness [2]. Oxygen therapy, corticosteroid therapy, and mechanical ventilatory support remain essential in the treatment of severe SARS-CoV-2 pneumonia [2]. Several vaccines are widely used to prevent COVID-19 infection, to stop the spread of the virus, and to reduce the rate of severe forms [3,4]. Vaccination has reduced the number of new cases, the length of hospital stay, and the rate of invasive mechanical ventilation [5]. Despite this, severe forms are still described with a winter resurgence (nearly 70,000 deaths in January 2022 and 40,000 deaths in January 2023). These epidemics are mainly related to the emergence of COVID-19 mutations and their viral variants presenting different characteristics from those of stem variants [2]. Thus, the emergence of new variants of SARS-CoV-2 threatens to wipe out advances in clinical research.

Low-dose radiotherapy (LD-RT) was used successfully during the first half of the 20th century in the treatment of a wide range of inflammatory and infectious diseases, including bacterial, viral, or inflammatory pneumonia [6]. This concept has been resurrected from the beginning of the pandemic by the radiotherapist community with several clinical trials conducted in different countries around the world. This review aims to describe the place of a historic therapeutic weapon in SARS-CoV-2 pneumonia management, represented by LD-RT.

## Review

Methodology

We performed this literature review using the PubMed search engine to identify the main articles that reported the place of LD-RT in the treatment of COVID-19 pneumonia. The MeSH terms used for the search were [COVID 19 OR SARS-CoV-2] AND [pneumonia] AND [low-dose] AND [whole lung radiotherapy OR lung radiotherapy]. The search was limited to full-text articles. The selection of articles was based first on the content of the abstracts and then on the content of the full text and clinical relevance. We included all trials that evaluated the role of low-dose radiotherapy in the management of severe pneumonia. We excluded from the review unpublished studies, abstracts without full articles, letters, and conference articles. The objective of this study is to review the range of data and clinical trials supporting the use of LD-RT in the management of COVID-19 patients.

Biological rationale of the LD-RT

SARS-CoV-2, an RNA virus, is responsible for CD4 T lymphocyte activation, pro-inflammatory macrophage recruitment, and interleukin release. This cytokine storm is the origin of severe forms of SARS-CoV-2 infection [7]. LD-RT was used successfully during the first half of the 20th century in the treatment of a wide range of inflammatory and infectious diseases. In their literature review, Calabrese and Dhawan reported a mortality reduction of 30% to 10% and rapid clinical improvement, with cure rates reaching 83% in patients with bacterial or viral pneumonia treated by LD-RT [6]. It should be noted that if irradiation with high doses has pro-inflammatory effects, LD-RT (less than 1 Gy) is responsible for anti-inflammatory action. This effect is due to complex mechanisms at different levels of the inflammatory process [8]. Alveolar macrophages play an important role in maintaining pulmonary homeostasis through immunosurveillance. Nerve and airway-associated macrophages (NAMs) are a subpopulation of interstitial macrophages identified in mice. They strongly express immunoregulatory genes, thus maintaining immune homeostasis by stimulating the secretion of immunosuppressive factors such as interleukin (IL)-10. However, their absence leads to the release of pro-inflammatory cytokines (IL-6, CCL2, CCL3, and CCL5) causing lung lesions. In a study conducted by Meziani et al., LD-RT (0.5-1 Gy) in mice reduced the rate of pneumonia owing to inflammation inducers (airways-instilled lipopolysaccharide). Indeed, they observed that even the use of LD-RT in vitro on human macrophages allowed an increase in the level of NAMs at the origin of the production of IL-10, involved in the fight against inflammation [9].

Other studies have demonstrated that LD-RT induces numerous changes in bronchoalveolar lavage fluid and serum. These modifications affect both markers of inflammation (cytokines) and immune cell mediators. LD-RT causes the early release of transforming growth factor-β, a regulatory cytokine involved in the initial phase of an anti-inflammatory process whose main role is to maintain immune homeostasis. in addition, the LD-RT anti-inflammatory effect lies in inhibiting immune cell infiltration into sites of infection or inflammation, suppressing primary cytokines production, and increasing the number of M2 macrophages [10].

Clinical rationale of LD-RT

Since the beginning of the pandemic, several clinical trials have been launched in different countries. The first trial that assessed the impact of LD-RT on COVID-19 pneumonia was the Rescue 1-19 trial from Emory University Hospital. It was a phase I-II trial that evaluated the safety and effectiveness of LD-RT in the entire lungs of seven patients with a median age of 90 years and multiple comorbidities. Five patients received a single fraction of low-dose irradiation (1.5 Gy) to both lungs. After 24 hours of radiation therapy, 60% of patients switched from supplemental oxygen to room air, and 80% of patients experienced radiological improvement. While another patient (80% overall recovery) was weaned from oxygen at 96 hours. The mean clinical recovery time was 35 hours. The irradiation resulted in rapid improvement in respiratory distress, neurological status, and radiographic infiltrates without acute toxicity. Based on these results, the Emory team concluded that the potential benefits of LD-RT should be explored further [11]. Then, in 2021, they published the results of this trial (Rescue 1-19) on the effectiveness of LD-RT at 28 days. They compared 10 non-intubated, oxygen-dependent patients who received LD-RT versus 10 other patients placed on standard treatment alone. The average time from admission to recovery was 10 days in the LD-RT arm versus 13 days in the control arm (p = 0.13). The average length of hospitalization was 19 days versus 22.6 days (p = 0.53). The average number of hospital days on supplemental oxygen, regardless of duration, was 13.1 versus 14.7 days. There was no difference in overall survival at 28 days between the two groups. However, significant improvement in biological disturbances in the LD-RT arm was noted [12]. The same team from Emory University Hospital published the results of this trial on a larger number of patients (40 patients). The main objective of this trial was to determine the safety and effectiveness of LD-RT administered concomitantly with other treatments (dexamethasone and/or remdesevir). LD-RT significantly improved markers of inflammation (C-reactive protein (CRP)) as well as markers of cardiac distress but had no significant impact on intubation-free survival [13].

In Iran, several studies have been performed. The first pilot study was conducted at Imam Hossein Hospital in patients over 60 years old hospitalized for severe pneumonia due to SARS-CoV-2. It evaluated the effectiveness of a 0.5 Gy versus 1 Gy LD-RT associated with the standard national treatment of COVID-19 [14]. With an 80% response rate in irradiated patients, the results of this study encouraged further studies in Iran, especially the one conducted at Imam Khomeini Hospital Complex. It was a non-randomized study that included 11 patients with severe refractory pneumonia. The study’s primary endpoint was improvement of chest X-ray severity score (CXRS), and the secondary endpoints were changes in mean oxygen saturation and 28-day mortality. The CXRS was significantly lower in the LD-RT group (p = 0.016). The mean oxygen saturation showed insignificant improvement in the first 24 hours after radiotherapy. In addition, the overall survival after 28 days was 32% in the LD-RT group versus 11% in the control group (p = 0.48). The study concluded that even low-dose whole-lung radiotherapy could improve the CXRS and oxygen saturation in seriously ill patients with COVID-19. Meanwhile, larger studies are required to determine the LDRT impact on mortality [15].

The Spanish Lowrad-Cov19 trial also evaluated the radiological response after lung irradiation at a dose of 1 Gy in one fraction among patients aged over 50 years old on corticosteroids. The investigators noted stability or even significant radiological improvement between the first and third scans (performed after seven days). After 112 days of follow-up, of the nine patients treated, seven were discharged from the hospital and two died [16]. Final results published in 2022 from this trial of 41 patients concluded that LD-RT was a feasible and well-tolerated treatment, with faster clinical improvement [17]. Despite the encouraging results of these studies, the Swiss Covid-RT-01 trial, which included a large number of patients, presented disappointing results. It was a randomized, double-blind, phase II study conducted among 22 patients intubated or under non-invasive ventilation, after 1 Gy lung irradiation, aiming to evaluate the reduction in mechanical ventilation time. It included elderly patients (median age of 75 years) with multiple comorbidities. This study showed no benefit of LD-RT on the primary endpoint (ventilator-free days) or overall survival at 28 days. Thus, LD-RT failed to improve clinical outcomes in patients with very advanced disease requiring mechanical ventilation [18]. Several trials followed with larger patient numbers to better place LD-RT in the therapeutic arsenal of COVID-19 pneumonia.

The IPACOVID trial included 100 patients with moderate-to-severe COVID-19 pneumonia with multiple oxygen-dependent comorbidities but were recused by intensive care units. Fifty patients in the experimental arm received a single dose of 0.5 Gy irradiation to both lungs. The main objective of this study was an improvement of at least 20% of the PaO_2_ or SpO_2_ ratio on FiO_2_. The results of this trial confirmed that LD-RT was a therapeutic alternative in patients with severe comorbidities. Even if the main objective was not achieved, the post-hoc analysis confirmed that LD-RT allowed an improvement in respiratory parameters at one week and one month, a reduction in hospital stay with a significant improvement in the LD-RT arm overall survival (log-rank, p = 0.027) [19].

In India, several studies have been conducted. WINCOVID was a phase II, randomized trial including COVID-19-positive patients with moderate-to-severe pneumonia (SpO_2_ <94% on room air, respiratory rate >24 breaths/minute, and SpO_2_/FiO_2_ (SF ratio) between 89 and 357). Initial results on 25 patients concluded that LD-RT was effective (clinical and oxygenation improvement at 10 days) and well tolerated by all patients [20]. Afterwards, the final results of this trial which randomized 51 patients into two groups were published. The experimental group of 34 patients received a single 0.5 Gy fraction on both lungs in addition to standard pharmacological treatment while 17 patients were placed on standard pharmacological treatment alone. Compared to the control group, patients who received LD-RT showed significant improvement in oxygenation, early clinical recovery time, early hospital discharge, and even radiological improvement. However, there was no significant difference in overall 28-day survival between the two groups. According to the study, LD-RT may be proposed in well-selected patients with moderate-to-severe oxygen dependence [21]. This trial presents impressive results supporting the effectiveness of LD-RT in the treatment of moderate-to-severe COVID-19 pneumonia. However, several criticisms may be raised, in particular, the lack of precision in patients’ vaccination status, with a difference between the two groups in terms of comorbidities. The IMpaCT-RT Study, an Indian trial that only included moderate pneumonia, had the primary objective of assessing the impact of LD-RT on preventing the progression from moderate pneumonia to severe pneumonia. The investigators randomized 13 patients into two groups (seven patients in the LDRT arm and six patients in the control arm). In the LD-RT arm, they observed an improvement in the SF ratio on D3, and a reduction in serum levels of lactate dehydrogenase, CRP, ferritin, and D-dimer on D14 but without a statistically significant difference between the two groups. The analysis of the National Early Warning Score 2 score showed an improvement in irradiated patients, but this improvement did not reach significance. Thus, LD-RT seems to prevent the progression of moderate pneumonia to severe pneumonia [22]. Another Indian study recently published by Dinakar et al. also evaluated the effectiveness of LD-RT in the management of COVID-19-positive patients with mild-to-moderate acute respiratory distress syndrome. They randomized 65 patients (33 in the experimental arm and 32 in the control arm) and concluded that LD-RT reduced the rate of unfavorable pneumonia after 28 days of LD-RT without added toxicity [23].

In the Mexican RTMX-20 study which included 60 patients (31 patients in the LDRT arm and 29 patients in the control arm), the mortality rate was estimated at 27.5% in the LD-RT arm and 58.6% in the control arm (p = 0.05). This trial compared mortality according to the severity of lung injury. The survival of patients with moderate respiratory damage was significantly better in the LD-RT arm compared to the control arm (100% vs. 40%, respectively; p = 0.01); however, they did not observe a significant difference in survival between the two groups in patients with severe respiratory compromise [24].

An African study aimed to evaluate the safety, feasibility, and toxicity of LD-RT in patients with severe COVID-19 pneumonia. The study included 10 patients, 60% of whom were discharged, and avoided intubation and mechanical ventilation in the majority of patients, seven days after LDRT. It was noted that four patients died following progression [25].

A recently published Polish study, which included 15 patients with severe pneumonia and multiple comorbidities, also concluded that LD-RT is feasible without increased toxicity. Improvements in SaO_2_ and SpO_2_ at 24 hours after LD-RT with a significant decrease in CRP (107.9 vs. 51.4, p = 0.007) and IL-6 (98.7 vs. 26.9, p = 0.006) were observed [26].

Table 1 summarizes the major clinical trials of LD-RT in the treatment of COVID-19 pneumonia published to date.

**Table 1:** Major clinical trials of LD-RT in the treatment of COVID-19 pneumonia. LD-RT = low-dose radiation therapy; LDH = lactate dehydrogenase; CRP = C-reactive protein

Clinical trial	Trial design	Number of patients	Mean age (years)	Primary endpoint	Radiation therapy scheme	Results
RESCUE 1-19 trial États-Unis 2020 NCT04366791 [11]	Phase I/II, not randomized	7	90	Clinical response (output hospitalization/withdrawal in oxygen), radiological response, and biological response	1.5 Gy to whole lungs	Four patients were weaned from oxygen at an average duration of 1.5 days. 80% of biomarkers were stable or improving after three days. The average discharge from the hospital was 12 days
RESCUE 1-19 trial États-Unis 2021 NCT04366791 [12]	Phase I, II	10: LD-RT; 10 control patients blindly matched by age and comorbidities	Median age: 78 (43–104)	Time to clinical recovery, radiographic improvement, and biomarker response	1.5 Gy whole-lung LD-RT	Median time to clinical recovery: 12 days in the control cohort and three days in the LD-RT cohort (hazard ratio = 2.9, P = 0.05). Statistically significant reductions in biomarkers
RESCUE 1-19 trial États-Unis 2021 NCT04366791 [13]	Phase II	20: LD-RT cohort; 20: matched controls	63 (49–88)	Safety and efficacy of LD-RT delivered concurrently with dexamethasone and/or remdesevir	1.5 Gy whole-lung LD-RT	Intubation rates: 14% with LD-RT, 32% without (p = 0.09). Biomarkers of inflammation (C-reactive protein, p = 0.02) and cardiac injury (creatine kinase, p < 0.01) declined following LD-RT compared to controls
Ameri et al. (2020) NCT04390412 Iran [14]	Pilot study	5	71.8 (60–84)	Change from baseline blood oxygenation (in terms of oxygen saturation), the number of hospital/intensive care unit stay days, and the number of intubations performed after radiation treatment	0.5 Gy in a single fraction	Mean time to discharge: six days for three patients. No acute radiation-induced toxicity
Darzikolaee et al. (2021) IRCT20170211032494N3 Iran [15]	Non-randomized controlled clinical trial	11: LD-RT group; 12: control group	55.2: LD-RT group; 51.4: control group	Improvement in the chest X-ray severity score (CXRS)	1 Gy dose to both lungs	The CXRS was significantly lower in the LD-RT group (8.7) compared to the control group (12.3) (p = 0.016)
WINCOVID trial, 2021, India [21]	Prospective, randomized, parallel-group, active-controlled trial	51 patients. 34: LD-RT group; 17: control group	40–90	Comparison of the efficacy of LD-RT based on an improvement in SpO_2_/FiO_2_ (SF) ratio, at 48 hours, 72 hours, 7 days, and 14 days from the time of intervention (LD-RT in LD-RT group and the first steroid dose in the control group)	0.5 Gy in a single fraction to whole lungs	LD-RT significantly improved oxygenation, time to clinical recovery, hospital discharge, and radiological resolution compared to the control group. No statistically significant in the 28-day mortality rate between the two groups
IMpaCt-RT Study, 2022, India [22]	Prospective, double-arm, interventional, randomized controlled trial	7: radiation arm; 6: control	56 (52–68)	To evaluate changes from moderate-to-severe pneumonia with the National Early Warning Score (NEWS-2) on days 3, 7, and 14	0.7 Gy to bilateral lung fields in a single fraction	LD-RT improved the outcome of SpO_2_/FiO_2_ on day 3, but it did not convert into a statistically significant improvement for the NEWS-2 score. The serum levels of LDH, CRP, ferritin, and D-dimer were significantly reduced after 14 days in the LD-RT arm
Dinakar et al. (2023), India [23]	Single-institute, randomized (1:1), open-label, parallel-group study	33 patients: experimental arm; 32: control arm	53.7 ± 8.8	Progression to severe disease (PaO_2_/FiO_2_ ratio)	0.5 Gy	Unfavorable outcome: 5 (15.2%) patients in the experimental arm, and 12 (37.5%) patients in the control arm, (p = 0.04)
LOWRAD-Cov19, Spain [17]	Prospective phase I-II	41	71 (60–84)	A radiological response assessed as severity and extension scores at days +3 and +7	A single fraction of 1 Gy to whole lungs	The extension score improved significantly (p = 0.02) on day +7. The mean baseline extension score was 13.7 (SD = ±4.9) with a score of 12.2 (±5.2) on day 3 and 12.4 ± 4.7 on day 7. No differences in the severity score
COVID-RT-01, Suisse [18]	Randomized, double-blind, phase II study	22	75	Ventilator-free days (VFDs) at day 15 post-intervention. VFDs were defined as the number of days a patient was alive and free of mechanical ventilation	1 Gy in a single fraction	No difference in 15-day VFSs was observed between groups (p = 1.00)
IPACOVID trial, Spain [19]	Multi-center, non-randomized, prospective trial	50 patients: LD-RT treated (experimental cohort); 50 patients: controls	85	The difference in the variation of the PaO_2_/fraction of inspired oxygen (PAFI) between the experimental and the control cohorts was measured 48 hours after LD-RT treatment (improving PAFI values by over 20% at 48 hours)	0.5 Gy in a single fraction	PAFI values did not improve. On post-hoc analysis, significant long-term respiratory improvement was noted. Increase in SpO_2_ (97% vs. 94%; p = 0.0052) and reduction in the FiO_2_ (27% vs. 35%; p = 0.038) after one week
RTMX-20 trial, 2022, Mexico NCT04534790 [24]	Matched, prospective, comparative cohort study	60 patients. 31: LD-RT group; 29: control group	LD-RT group: 53 (27–87); control group: 57 (36–87)	Survival in patients treated with LD-WLI	A single dose of 1 Gy to both lungs	Mortality: 27.5% in the radiotherapy group compared with 58.6% in the control group (p = 0.05)
Saleh et al. (2022), Kenya [25]	Pilot Ib/II investigator-initiated, single-center trial	10	59	Safety/feasibility, and toxicity within the first 24 hours post-LD-RT	A single fraction of 1 Gy to the whole lung	90% of patients avoided mechanical ventilation within seven days of LD-RT. No acute toxicity at 24 hours following LD-RT
Rutkowski et al. (2023), Poland [26]	Phase II	15 patients	Median 66 (range 49–78)	NA	1 Gy to the bilateral lungs	28-day mortality was 13%. Median oxygen saturation improved within 24 hours after LD-RT in 14/15, with median SpO_2_ values of 84.5% vs. 87.5% (p = 0.016), respectively. Significant decline in CRP and IL-6 within 24 hours after LDRT

Limitations and risks of LD-RT

In summary, many limitations should be discussed. First, the irradiation dose used was not uniform in the different published trials (between 0.5 and 1.5 Gy). Second, the number of patients included in the majority of trials was limited. Finally, the medical treatment received was not always mentioned.

The risk of secondary cancer after radiation therapy has been discussed in several studies. The dose range of LD-RT, which is well below the threshold for deterministic pulmonary effects (typically 20 Gy in conventional fractionation) cannot cause any toxicity. The absolute stochastic excess risk of radiation-induced secondary cancer over 20 years is 0.4% and 1.2%, respectively, for a dose of 0.5 Gy and 1.5 Gy. The magnitude of risk is so low, particularly for elderly patients, that the potential treatment benefit would far outweigh the risk of secondary cancer [27].

This risk of radiation-induced secondary cancer is very low, especially in cases of a real risk of death from SARS-CoV-2 pneumonia, and the advanced age of patients at this risk would make such concerns irrelevant [28]. According to the study of Kirsch et al., the risk of cancer due to chest X-ray exposure varies according to sex, age, and radiation dose. This risk decreases significantly with age but remains less well-established in young patients. However, the majority of published trials mainly included elderly patients. The single dose prescribed in pulmonary LD-RT is low and remains even lower than the natural radiation doses to which the inhabitants of the city of Ramstar in Iran were exposed, which was 260 mSv. This radioactivity came from radium dissolved in the city’s thermal waters. However, the cancer rate of Ramsar residents was not significantly different from that of the population living in less exposed areas [29].

The second risk mentioned in the literature is viral reactivation with the risk of selecting mutated viral stems following the damage that radiotherapy can cause to viral RNA. According to Rodel et al., low-dose radiation of 1 Gy delivered in SARS-CoV-2 pneumonia cannot induce viral reactivation, contrasting with antiviral treatments causing more selective pressure on the virus [30].

## Conclusions

The incidence and especially the severity of COVID-19 infection has significantly decreased since the advent of vaccines. However, some severe forms are still described. LD-RT remains an alternative to offer in well-selected patients. In light of the various trials published to date, LD-RT appears to be able to reduce the progression of moderate COVID-19 pneumonia to severe pneumonia. Therefore, LD-RT remains safe and feasible showing a promising place in patients who progress on standard systemic treatment, as well as in limited-income countries where therapeutics are not always available. However, further randomized clinical trials including a larger number of patients are needed to justify the LD-RT definitive role in the management of COVID-19 pneumonia and to define the profiles of patients who will benefit from it.
